# Use of Geographic Information Systems in Physical Activity Interventions: a Systematic Review

**DOI:** 10.1097/pp9.0000000000000022

**Published:** 2019-06-14

**Authors:** Liliana Aguayo, Mariha Khan, Reynaldo De Leon, Andiara Schwingel

**Affiliations:** a Department of Preventive Medicine, Northwestern University Feinberg School of Medicine, Chicago, Ill.; b Mary Ann & J. Milburn Smith Child Health Research, Outreach, and Advocacy Center, Stanley Manne Children’s Research Institute, Ann & Robert H. Lurie Children’s Hospital, Chicago, Ill.; c Rush University Medical Center, Rush University Medical College, Chicago, Ill.; d Department of Health and Behavior Studies, Teachers College, Columbia University, New York, N.Y.; e Department of Kinesiology and Community Health, College of Applied Health Science, University of Illinois at Urbana-Champaign, Champaign, Ill.

**Keywords:** geographic information systems, physical activity interventions, physical geographic factors, social geographic factors, systematic review

## Abstract

**Introduction:**

The promotion of physical activity is inextricably dependent on the environment. This systematic review summarizes studies that used geographic information systems to account for the role of geographic features in the design, implementation, or evaluation of interventions that promoted physical activity.

**Methods:**

Pubmed, ProQuest/PsycInfo, and Cochrane Library were searched for physical activity interventions that employed geographic information systems. The search was conducted with an algorithm that included 10 geographic, 28 intervention, and 9 physical activity search terms. Data were systematically reviewed using a standardized form based on the PICOS framework (participants, interventions, comparison, outcomes, and study design). Quality of the studies included was independently rated on 14 criteria (Registration number CRD42016046011).

**Results:**

Search yield 12,518 published articles, of which, 19 studies satisfied our inclusion criteria. Proximity to recreational areas and neighborhood walkability were the most common geographic factors measured in studies of physical activity interventions. Interventions focused on supporting physical activity by providing recreational spaces and adequate infrastructure to participants. Fifteen intervention studies assessed socioeconomic environmental factors along with physical environmental factors. Support groups were introduced by 4 of the interventions to offset social environmental barriers in the geographical environment. Given the lack of consistency in measurements of physical activity, and long-term assessments, it was not possible to determine if findings are attributable to the geographic environment or a novelty effect.

**Conclusion:**

More research is needed to better understand the physical and social factors within the geographic environment that work as barriers or facilitators of physical activity changes.

## Abstract 2

Supplemental Digital Content is available in the text.

## Introduction

A third of adults and four-fifths of adolescents and children around the world, do insufficient physical activity to prevent obesity.^[1,2]^ Urbanization and technological advances have triggered many environmental changes associated with declines in physical activity linked to the worldwide rise in obesity rates.^[3–5]^ Urbanization over the past 3 decades has favored increased urban sprawl and car- oriented communities, whereas technological advances have reduced the physical demands of work and increased sedentary activity.^[3,4]^

Increasing physical activity, particularly among the most inactive and vulnerable populations is a public health priority listed in the “Global Noncommunicable Diseases Action Plan 2013–2020” and “Healthy People 2020.”^[6]^ The identification of geographic variations in health disparities suggests that physical activity cannot be solely attributed to individual factors. Efforts to prevent chronic diseases and reduce health disparities need to consider geographic barriers and opportunities for healthy lifestyles.^[6,7]^

Physical activity is inextricably dependent upon features in the geographic environment.^[8]^ Geographic physical and social characteristics of disadvantaged neighborhoods including lacking sidewalks and green areas, traffic safety, limited availability of healthy foods, land use, urban sprawl, and housing insecurity have been linked to increased rates of noncommunicable diseases and health disparities.^[3–6,9–12]^ These geographic attributes have been identified by several cross-sectional reviews as key barriers for physical activity.^[10,12,13]^ Yet, the benefits of using geographic information to guide physical activity interventions and empirical connections between changes to geographical environments and physical health outcomes are scarce.

An earlier systematic review that examined the effects of environmental and policy interventions aimed at increasing physical activity encouraged researchers to use objective measurements of the environment to account for geographic effects.^[14]^ In this earlier review, Sallis et al^[14]^ concluded methodological difficulties hindered the implementation of environmental approaches in physical activity interventions. Since then, technological advances have made geographic software programs more accessible for health practitioners. These improvements have enable researchers to analyze the influence of geographic factors on physical activity implementing diverse tools to layer geographic physical and even social information.

From its inception, Geographic Information Systems (GISs) were designed to traverse a variety of disciplines. The purpose of GIS is to layer diverse types of data over a geographical map to illustrate relationships with data and locations. GIS ability to address the multifaceted and complex relationship between temporospatial variables lends itself well to the study of health. In health studies, GIS has been essential to track the location and movements of diseases and has been a major tool in the control and study of communicable disease outbreaks.^[6]^ Unfortunately, beyond epidemiological and cross-sectional studies, the application of geographic information through GIS or similar methodological approaches has been limited.

In this review, we aim to systematically examine the implementation of geographic programs or GIS to include geographic information in the design, implementation, or evaluation of interventions that promoted physical activity. For the purpose of this review, geographic information will refer to any objective or subjective assessment of environmental factors with a spatial location, which can support, moderate, mediate, or inhibit physical activity. Whenever we are referring to objective measurements, we are including all measurements of physical activity or geographic information collected using a given instrument (pedometer, accelerometer, heart rate monitor), to systematically calculate the repetition of a given unit (ie, steps, bouts, minutes). In contrast, when we refer to subjective report tools, subjective instruments, or subjective measurements, we include assessments that relied on individual’s perceptions and recall abilities. Instead of being directly measured, subjective assessments are collected from individual-reporting using questionnaires which may or may not have undergone validation. Geographic factors examined as geographic information also include any changes in the built environment and any environmental factors for which location is assessed and considered to influence participation in physical activity.

The aim is to summarize the different approaches employed to operationalize geographic information in the promotion of physical activity. Findings from this review introduce empirically tested approaches that we hope will serve as a resource that informs clinicians, researchers, and stakeholders about options to benefit from geographic information.

## Methods

The protocol for this systematic review is registered in PROSPERO International Prospective Register of Systematic Reviews (Registration number CRD42016046011). Upon completion of the piloting of the study selection process, the protocol was amended to include participants of all ages, and variuos programs, including GIS, which have been used to examine geographic information.

### Search strategy

This literature review examined scientific articles describing or evaluating physical interventions published before April 2017. The review began with a systematic search in 3 databases: Pubmed, ProQuest/PsycInfo, and The Cochrane Library [Cochrane Database of Systematic Reviews, Cochrane Central Register of Controlled Trials (CENTRAL) Cochrane Methodology Register]. The first 2 databases were selected because of their wide breath of health information, and because our aim was to introduce this review to health professionals who may not necessarily be geography or urban planning experts, but are interested in uses of geographic information to promote physical activity. The search was conducted with an algorithm that included 47 search free text and Medical Subject Headings. Our search included articles that resulted from any combination of 3 terms, one term from each search category: (1) “Geographic Information” (10 terms); (2) “Health/Wellness interventions” (28 terms); and (3) “Physical Activity” (9 terms) (a complete list of search terms is available in **Supplemental Digital Content 1, *http://links.lww.com/PP9/A4***). Whenever possible, we limited our search to: (1) studies published after January 2000 (considering the commercial release of Esri’s ArcGIS software (Esri, Redlands, CA), which triggered the proliferation of diverse tools that enable researchers to analyze the influence of geographic factors on physical activity) and (2) human species only. The third database was selected to include bibliographies from similar systematic reviews. Bibliographies from similar systematic reviews were hand searched to identify additional studies that satisfied inclusion criteria. We only included publications available in (3) English and (4) published in peer-reviewed journals. Editorials, policy briefs, letters, and commentaries were excluded.

### Study selection

The search yielded 12,642 references. The search was conducted twice, first in November 2015, and updated in April 2017. After removing 1120 duplicates, the title search included 11,522 references, 8998 from the initial search, and 2524 from the updated search. To be eligible for inclusion, studies had to examine the promotion of physical activity alone or in combination with other lifestyle components (ie, diet); and use any geographic software or similar methodology to consider geographical information in the design, implementation, or analyses of the physical activity intervention. For eligibility purposes, geographic information referred to the assessment of the spatial location of any objective or subjective factors considered to support, moderate, mediate, or inhibit physical activity.

One researcher completed an initial screen of article titles. The title review yielded 1614 references that fit the inclusion criteria or were otherwise unable to be deemed irrelevant based on the title alone. Examples of studies eliminated in this stage included cross-sectional studies, animal studies, or studies not available in English.

**Fig 1.**
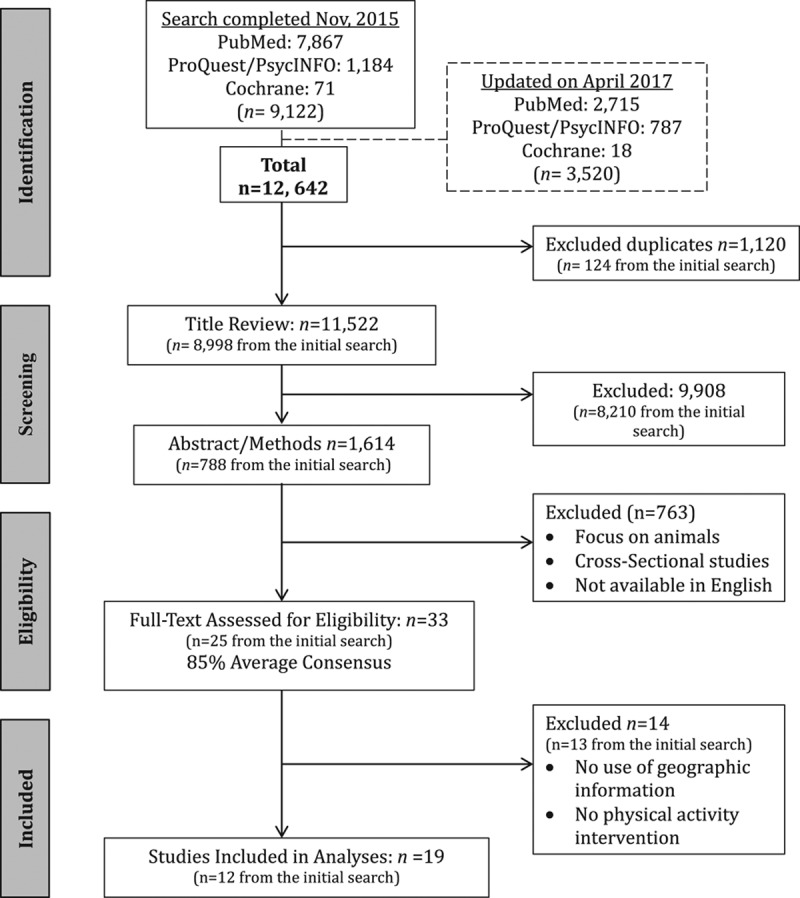
PRISMA flowchart of search strategy and study selection process. The flow diagram was adapted from PRISMA 2009 to describe the identified study records, and the exclusion criteria.

In the next step, 2 researchers screened 1614 references independently based on abstracts and methodology sections. Studies were reviewed based on methodology, specifically to ensure that geographic information was included. Systematic reviews were examined to determine if there were any additional studies that satisfied inclusion criteria. From these steps, researchers identified 34 studies that satisfied the inclusion criteria. Upon completion of the title, abstract, and methodology review, an agreement rate of 85% (28/33) was achieved among 2 reviewers. A third reviewer was included to complete full-text reviews of the 33 studies identified, and limit biases introduced by reviewers. Discrepancies were resolved by consensus among all reviewers. The final list included 19 studies (Fig 1). Data from all selected studies were extracted using a standardized form (**Supplemental Digital Content 2, *http://links.lww.com/PP9/A5***).

### Data extraction

A standardized form was created based on the PICOS framework (participants, interventions, comparison, outcomes, and study design) to systematically extract data.^[15]^ Information recorded from each study included author, publication year, study aims, setting of the intervention, country, description of participants, elements of the intervention (ie, physical activity of multiple components), study design, comparison group, outcomes, geographical factors reviewed and tools used to approach geographical environments, outcomes attributable to geographic information, length of intervention, and duration of follow-up. If any information needed to complete the standardized form was not available in the selected manuscripts, additional publications were reviewed, and if information was not found, or other publications were not available, corresponding authors were contacted.

### Assessment of geographic information

We used 4 different criteria to examine the different approaches to geographic information in physical activity interventions. First, we examined whether studies: (1) measured or (2) modified factors in the geographic environment, and then whether the interventions’ interactions with the environments addressed (3) primarily physical factors, or (4) if the location of social factors was also considered.

### Quality assessment

To assess the quality of the studies included in this review, interventions were independently rated on 14 criteria by 2 researchers. The rating scale developed was adapted from the Cochrane review tool, and from a scale designed to assess study quality of public health intervention studies.^[16,17]^

Criteria scored included: description of study aims, details of target population, report of attrition rates, description of the context where the intervention took place, description of field work, duration of the intervention, follow-up assessments and length of follow-up assessment, use of objective data to assess physical activity, use of subjective instruments to assess physical activity, use of validated assessment of physical activity, detailed description of data analyses, power analyses considerations, tailoring intervention to participants settings or culture, and acknowledgment of limitations. An additional point was added to the quality score of each study for additional follow-up assessments and for detailed descriptions of the intervention field work. Possible scores ranged from 0 to 17, with higher scores representing higher quality of evidence reported.

To reduce risks of bias in the review and quality assessment, 2 reviewers worked independently, and agreement was assessed by a third reviewer. Discrepancies were discussed in consensus with the third reviewer until agreement was reached among all reviewers.

## Results

A total of 19 studies meet inclusion criteria for this systematic review (Fig 1). The most common reason for exclusion of studies reviewed was the lack of geographic information, or cross-sectional approaches that did not promote changes in physical activity. Although 3 of the included studies did not report the name of the specific program used to assess geographic information,^[24,29,32]^ these studies were included because their methods to measure physical geographic features were described in detail, and presume the use of a geographic program or introduce a methodology that can be replicated using geographic information systems.

Six interventions were conducted in the United States.^[18–23]^ Ten interventions took place in Europe, specifically, 1 in The Netherlands,^[24]^ 2 in Denmark,^[25,26]^ 5 in the United Kingdom,^[27–31]^ 1 in Belgium,^[32]^ and 1 in Scotland.^[33]^ Two interventions were conducted in Australia,^[34,35]^ and 1 in Colombia.^[36]^ Although most interventions were conducted in developed countries, there was a lot of heterogenety in the key characteristics of the interventions that satisfied inclusion criteria (Table 1). The studies included in our review were published between 2003 and 2016, with 79% (15/19) published after 2012.

**TABLE 1.**
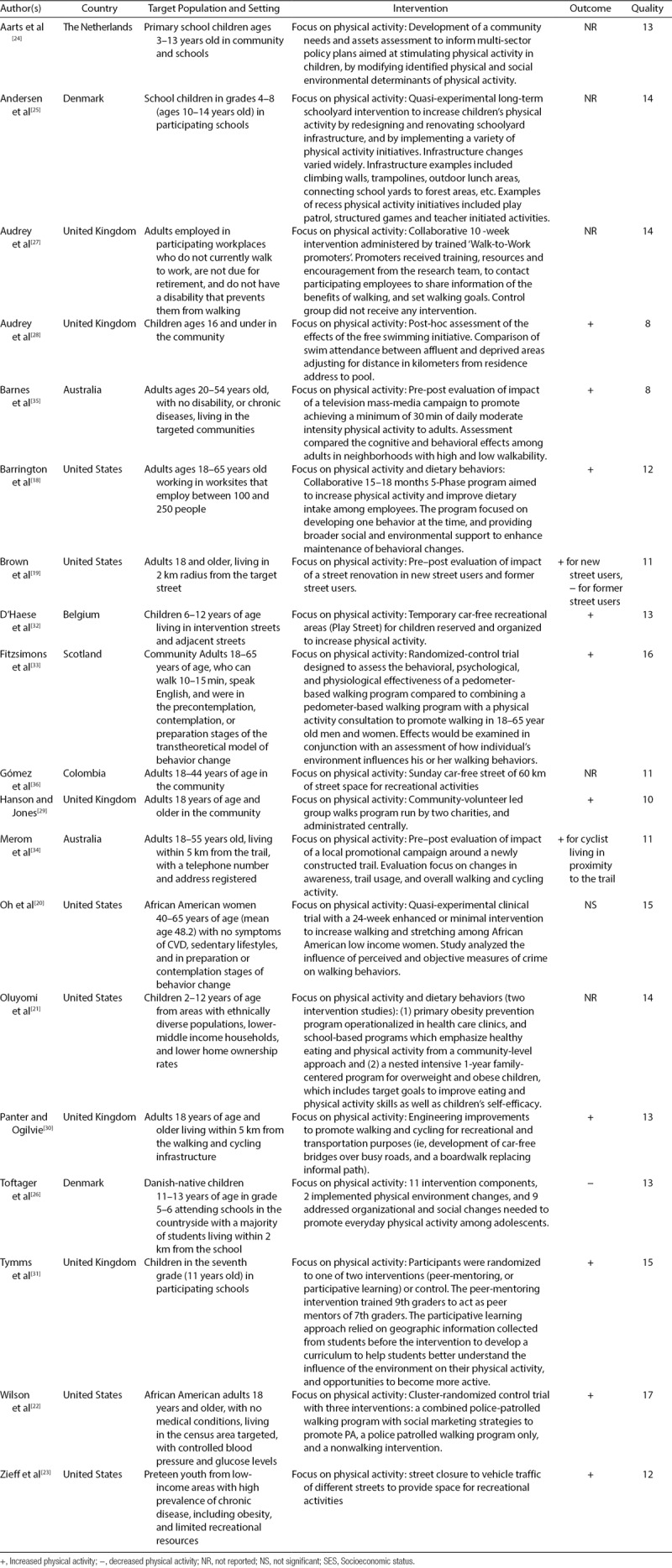
Key Characteristics of Physical Activity Interventions Included in the Review

### Applications of geographic information

Consistent with the focus of our search, all studies measured geographic factors that influence physical activity. In all interventions, measurements of geographic factors were done objectively through audit tools, coding indexes, and visualizations in GIS. In addition to these objective measurements of geographic factors, 2 studies (17%) examined subjective physical and social geographic barriers and facilitators of physical activity.^[26,35]^ Aarts et al^[24]^ measured parent’s reported perceived physical and social neighborhood characteristics, and Fitzsimons et al^[33]^ measured self-reported perceived geographic barriers or facilitators of physical activity.

Interventions that focused primarily on physical geographic factors aimed to provide spaces to encourage physical activity.^[21,32,36,37]^ Three interventions increased physical activity by modifying the infrastructure through construction projects (eg, street improvements, bridge construction, and trail development).^[19,30,34]^ Other physical activity interventions implemented physical infrastructure modifications through policy changes, which impacted the geographic environments by providing temporary access to recreational spaces. Examples of these policy approaches included “Play Streets,” and government-sponsored swimming sessions.^[23,28,32,36]^

Thirteen interventions included in this review (68%) used a geographical approach to investigate social factors that may influence physical activity (eg, crime, safety, income, and neighborhood socioeconomic status).^[18,20–29,32,33,36]^ Of the 13 interventions that considered the influences of social factors, 9 interventions (42%) implemented strategies to limit the negative impact of social barriers on physical activity (eg, peer support groups, police patrol walks).^[18,22–27,29,32]^ Six of these studies that modified social factors, organized trainer-led activities to enhance social support among the participants.^[18,22,26,27,29,32]^ Most of the interventions that enhanced social support yielded positive improvements in physical activity,^[18,27,30,32,38]^ but not all.^[26]^

### Physical and social geographic factors

We identified 8 interventions that assessed both, social and physical geographic factors (Table 2). Proximity was the most common physical geographic feature measured, followed by neighborhood walkability. Characteristics related to socioeconomic status were the most common social environmental factors that were measured and geographically examined as important correlates of physical activity. Within the examinations of socioeconomic status, 7 intervention studies addressed the neighborhood socioeconomic status, as opposed to the individual’s socioeconomics level,^[18,22,23,28,29,32,36]^ and 2 interventions complemented their assessments by analyzing physical geographic factors with the location of neighborhood crime.^[20,22]^

**TABLE 2.**
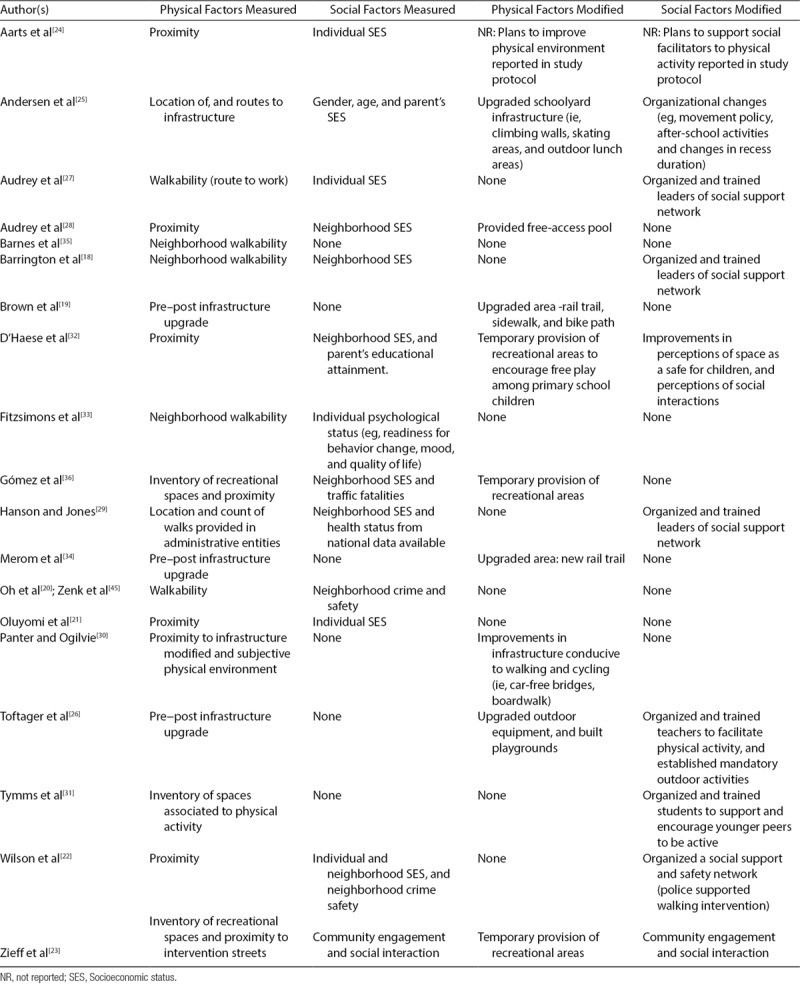
Physical and Social Environmental Factors Addressed in Physical Activity Interventions

All interventions that modified social environmental factors to support physical activity did so by organizing support groups within worksites or the community settings. In addition to fostering a support group, Wilson et al^[22,38]^ introduced police-patrolled walks as an innovative approach to effectively offset the objective and subjective safety concerns previously identified as an environmental barrier to physical activity in the target community.^[22,38]^ Although police-patrolled walking interventions introduce an innovative approach to address concerns of neighborhood safety, participation in walking interventions was only marginally affected by crime-related safety approaches.^[20,38]^

### Physical activity approaches

Different modes of physical activity were promoted (Table 3). Ten interventions (53%) focused on increasing physical activity and limiting sedentary behaviors by encouraging participants to limit sedentary activities and promoting sports participation, outdoor play, and active transportation.^[18,19,21,23–26,31,32,36]^ Five interventions promoted walking (26%),^[20,22,27,29,33]^ 3 interventions promoted walking and cycling (16%),^[30,34,35]^ and 1 intervention promoted swimming (5%).^[28]^

**TABLE 3.**
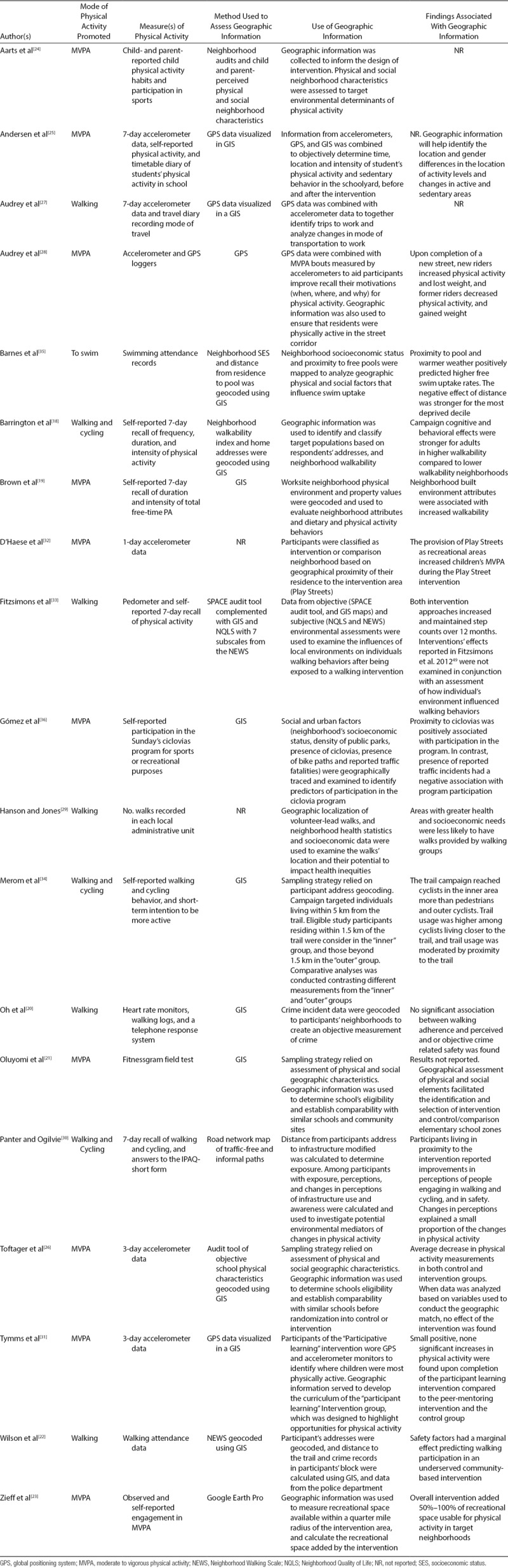
Details of Measurements and Applications of Geographic Information in Physical Activity Interventions

All studies measured physical activity, 6 (32%) used objective measurements.^[19,21,25–27,31]^ Seven interventions (37%) used self-reported, subjective assessments,^[18,23,24,30,34–36]^ or proxy measurements, like attendance data.^[22,28,29]^ A combination of objective and subjective measurements of physical activity was used in 3 studies.^[20,32,33]^ Of the 9 studies that collected objective measurements of physical activity, 6 (25%) used accelerometers,^[19,25–27,31,32]^ 1 intervention (8%) used pedometers,^[33]^ 1 (8%) used Fitnessgram (a fitness field test; The Cooper Institute, Dallas, TX),^[21]^ and 1 used heart rate monitors.^[20]^ Subjective measurements of physical activity used included researcher observations with a validated tool,^[23]^ and different self-reported validated instruments that assessed frequency, intensity, and duration of physical activity. Studies that combined objective and subjective approaches relied on pedometers with self-reported 7-day recall,^[33]^ data collected using heart rate monitors and walking logs,^[20]^ and 3-day accelerometers data complemented by answers from self-reported questionnaire of children’s wellbeing.^[31]^

With the exception of one study^[24]^ on multisector policy intervention, all interventions included had been administered and completed at the time of our review. Yet, 4 of the intervention studies reviewed had not yet published all available results,^[21,25,27,37]^ and 2 had published only results of data collected during the pilot assessments.^[23,32]^

### Participants

There was great diversity among the populations targeted by the interventions examined (Table 1). Eight interventions (42%) targeted children 2–16 years of age.^[21,23,25,26,28,31,32,37]^ Eleven interventions (58%) targeted adults 18 years of age and older,^[18–20,22,27,29,30,33–36]^ 2 of which targeted older adults (8%).^[20,38]^ Out of the 19 interventions, 9 (47%) included more than 1000 participants,^[18,21,23,24,26–28,30,31]^ and 10 (53%) interventions included between 79 and 939 participants.^[19,20,22,25,29,32–36]^

Seven interventions (37%) focused on promoting physical activity among specific racial or ethnic groups.^[20–23,26,29,36]^ Four of these interventions that targeted racial or ethnic minorities were conducted in the United States, 2 of them exclusively focused on African Americans,^[20,22]^ and 2 on participants living in ethnically diverse disadvantaged neighborhoods.^[21,23]^ A fifth intervention conducted in an ethnically diverse community took place in Cali, Colombia.^[36]^ Although the authors did not specify whether the neighborhood setting was economically disadvantaged, the program reported the inclusion of an ethnically diverse, socially disadvantaged community.^[36]^ The sixth intervention targeting a specific ethnic or racial group was conducted in Denmark. This study focused exclusively on schools where the majority of students were native Danish.^[39]^

A national walking program implemented in the United Kingdom included a diversity of ethnic and racial groups.^[29]^ However, the analysis of this walking program demonstrated this physical activity intervention had limited participation from non-White minorities, and significantly less walks were offered in ethnically diverse and disadvantage areas.^[29]^

### Study design

A quasi-experimental controlled research design was utilized by 8 (42%) interventions.^[20–22,25,27,31,33,39]^ Eight of the interventions relied on a pre–post design,^[18,19,23,24,30,32,34,35]^ and 3 used a post-hoc comparison between groups to examine intervention effects.^[28,29,36]^ All studies (79%) collected quantitative data, 4 of these collected both quantitative and qualitative information (21%).^[24,25,27,33]^ Seventeen interventions (89%) focused exclusively on increasing physical activity among participants,^[19,20,22–25,27–36,39]^ with only 2 multicomponent interventions (11%) that aimed to increase physical activity and improve nutritional habits.^[18,21]^

There was great heterogeneity among measurements of physical activity, characterizations of geographical environments, and primary outcomes reported (Table 3). Physical activity was the primary outcome of 14 (74%) interventions.^[19,20,22,23,25,27–33,36,39]^ Two interventions (11%) focused primarily on weight-related measurements,^[18,21]^ 2 (11%) on campaign awareness,^[34,35]^ and one ongoing study had not published information regarding their primary outcome by the time our review was completed.^[24]^

### Quality assessment

Overall, study quality was high. Quality scores ranged from 8 to 17, with 17 being the highest possible score, and a mean score of 12.6 ± 2.4 (Table 1). The criteria most often lacking were power analysis considerations, which were reported by 37% (7) of the interventions.^[19,21,24,26,27,31,33]^ Follow-up and long-term assessments were planned or conducted in only 47% (9) of the intervention studies reviewed.^[20–22,25,27,30,31,33,36]^

### Intervention settings

The interventions were delivered through a variety of settings such as: workplace, schools, and neighborhood/community, with a large majority of them using a community or neighborhood based approach. Of the 11 interventions that targeted adults (58%), 2 were implemented in worksites (11%),^[18,27]^ and 9 recruited adults from the community or neighborhoods targeted (47%).^[19,20,22,29,30,33–36]^ Among the 8 physical activity interventions that targeted children primarily, 3 were school based (16%),^[25,26,31]^ 3 were community based (16%),^[23,28,32]^ and 2 included multiple settings in their communities with schools as focal points supported by primary healthcare clinics^[21]^ and by multisector approaches.^[24]^

### Work-based interventions

Two of the 19 interventions targeted adult populations in different worksites. The “Walk-to-Work,”^[27]^ and Promoting Activity and Changes in Eating (PACE)^[18]^ physical activity interventions shared a multicentered cluster randomized-control trial design. In the “Walk-to-Work” intervention geographic information was collected to separate walks to work from other walks and evaluate the effects of the intervention that promoted walking to work.^[27]^ The PACE intervention implemented a geographic inventory of physical activity opportunities, and food services available in the 34 worksite neighborhoods where the intervention was administered.^[18]^ In addition to promoting increasing walking rates among employees, the PACE intervention also promoted increasing other modes of physical activity (ie, run, exercise classes, etc.), and improving dietary habits.^[18]^ The geographic information from the neighborhood inventories was used to examine the role of availability of physical activity opportunities, and food services, in relation to behavior changes (walking and fruit and vegetable intake).^[18]^ Disparities in worksite neighborhoods explained intervention effects.^[18]^ Worksites in more affluent neighborhoods supported improvements in physical activity and healthy eating, whereas underserved areas were associated with lower frequency of walking and lower fruit and vegetable intake.^[18]^

### Community/neighborhood-based interventions

Community settings were considered in 63% of the physical activity interventions that measured or modified physical geographic factors. Community approaches that targeted children were all policy-driven changes with a wide range of complexity. One community approach examined the geographic and social differences in the effects of a policy initiative that provided swimming sessions to children under the age of 15.^[28]^ Two studies examined the impact of “Play Streets” on physical activity changes among children^[23,32]^ and a third “Play Streets” study examined physical activity effects among children and adults.^[36]^

“Play Streets,” or “ciclovias,” introduced a novel policy approach to address geographic structural or financial barriers that may limit the provision of recreational spaces in park-deprived neighborhoods. In these physical activity interventions, streets in neighborhoods with limited recreational spaces were temporarily assigned to be car-free play areas for children and adults.^[23,32,36]^ Three of the studies included in this review reported positive effects on the physical activity of children and adults who participated in “Play Streets” programs in the streets of Colombia,^[36]^ Belgium,^[32]^ and the United States.^[23]^ In addition to providing car-free play zones, these approaches introduced recreational equipment and activities to support the promotion of physical activity. Examples of equipment included a box with play equipment,^[32]^ a climbing wall, and bicycle ramps,^[23]^ and recreation zones, with “aerorumba” (aerobic sessions with Latin dance rhythms).^[36]^ Studies assessing the effects of “Play Streets” found that these programs yielded positive physical activity changes among children and adult participants,^[23,32,36]^ and improved social participation in the community.^[23,32]^

Other community-based interventions included in this review examined the geographic differences in cognitive effects of physical activity campaigns^[34,35]^; increments in physical activity attributable to changes in the built-in-environment after infrastructure modifications,^[19,30,34]^ and outcomes resulting from walking programs.^[20,22,29,33]^

Access and proximity to walking infrastructure were found to positively influence cognitive effects of physical activity campaigns.^[34,35]^ Merom et al^[34]^ examined the influence of geographic factors on the effects of a physical activity campaign, and the physical activity outcomes associated with infrastructure improvements. Although proximity was found to positively influence cognitive effects and yield increases in cycling, remoteness not only was associated to lower awareness of the physical activity campaign but also to decreases in cycling.^[34]^

Three studies examined physical activity changes attributable to changes in the built-in-environment after infrastructure improvements.^[19,30,34]^ In all 3 studies, increases in the amount of time spent walking and cycling were associated with the use of the new infrastructure, which benefited mostly residents living in proximity to the enhanced routes.^[19,30,34]^

Among the interventions included in this review, walking was the only physical activity promoted by organized facilitators. Three quasi-experimental research interventions based on community designs introduced facilitator-led group walks among underserved communities,^[20–22,33]^ and a fourth study examined the social differences in the provision of walks hosted by a national non-for-profit walking program.^[29]^ These studies found that although social life and income are important predictors of participation in organized walking groups,^[22]^ greater need is not naturally associated with greater provision of services.^[29]^ As noted by Hanson and Jones,^[29]^ unless measurements are set in place to promote equity, physical activity interventions can lead to greater inequity by benefiting more active, and wealthier individuals.

### School-based interventions

There was great heterogeneity among 5 school-based interventions included in this review, yet all school-based interventions assessed existing physical geographic factors of the environment before the implementation of their interventions. One study used the geographic information collected during the formative stage to inform lessons on places that are most conducive to physical activity among targeted children.^[31]^ Two studies used physical and social geographic information primarily to improve their sampling strategy by considering geographic physical and social factors from schools as control or comparison sites during the match-pair design of intervention evaluation.^[21,26]^ One study used the geographic information to assess if changes in the infrastructure of the environment had triggered changes in the places where children are physically active.^[25]^ Last, one intervention did not provide specific details about the uses of geographic data beyond their plans to rely on policy changes to modify social and physical environments.^[24]^

All of the school-based interventions proposed changes to the social (organizational) environment to increase physical activity among children, with 3 school-based programs also proposing to implement improvements in the physical environment, particularly in the infrastructure of the playground areas.^[24–26]^ Among the 3 interventions that proposed physical environmental improvements, only the intervention by Toftager et al^[26]^ had published outcomes from the intervention. In this assessment, although all participating schools upgraded their playground areas and implemented sport programs, no significant changes in physical activity behaviors were found among children ages 11–13 years of age.^[26]^

The social environmental changes introduced to promote physical activity in schools included peer-mentoring interventions,^[26,31]^ teacher-led physical activities,^[21,26]^ and 2 physical activity interventions that focused on policy changes, such as recess length regulations, and after school policies.^[24,25]^

One peer-mentoring approach, complemented peer mentoring with improvements in infrastructure and other organizational changes that supported physical activity in schools.^[26]^ The other peer-mentoring model matched children ages 11 and 12 years old with older peers (ages 13–14 years old).^[31]^ In this intervention, older peers were instructed on how to encourage and monitor physical activity of their younger peers.^[31]^ The peer-mentoring approach was implemented by itself in one subsample.^[31]^ A second subsample examined a peer model complemented with geography lessons.^[31]^ In the geography lessons, children learned about places identified during formative research to be conducive to physical activity.^[31]^ Modest physical activity improvements were found among participants of the peer-model intervention that was complemented by geographic lessons. None of the peer-mentoring programs yielded significant physical activity changes.^[26,31]^

## Discussion

The usefulness of geographic information in the promotion of physical activity is becoming increasingly clear. All intervention studies examined acknowledged the importance of geographic factors to physical activity by either measuring resources available (walkability and inventory of recreational spaces),^[18,20,23,27,29–31,33,35,36]^ measuring the distance to resources (proximity),^[21,23,24,28,32,36,38]^ or by providing spaces that support physical activity in areas with limited opportunities for physical activity.^[19,23–26,28,30,32,34,36]^ Across the studies reviewed, it was consistently observed that infrastructure improvements alone do not lead to sustainable changes in physical activity if perceptions of the neighborhood are not modified, or awareness is low.

The development of active-friendly neighborhoods needs to consider the resources and barriers in their surroundings, and the different ways in which geographic factors impact the perceptions and behaviors of communities. An environment conducive to physical activity needs to be one where not only physical infrastructure is available but also where the social context provides a supportive setting. Although “free play” was the activity most often promoted to increase children’s physical activity,^[23–26,31,32,36]^ walking programs were most often organized by interventions targeting adults.^[20,29,33,38]^ Free play and walking programs inherently combined physical and social geographic factors.

In an umbrella review of systematic reviews, Bauman et al^[1]^ observed that the study of geographic factors associated to physical activity is well advanced. However, most studies of geographic factors associated with physical activity are cross-sectional, and the implementation of physical activity interventions that verify the causal role of geographic factors remains scarce.^[1]^ In this systematic review, we documented a wide variety of approaches that researchers implemented in different stages of physical activity interventions to account for the effects of a variety of objective and subjective geographic factors. Although distance, traffic speed, density, diverse housing types, and mixed land use have been documented to influence walking and cycling in previous systematic reviews of cross-sectional studies,^[1,40,41]^ this systematic review adds to the literature by summarizing the available causal evidence of physical activity changes achieved by addressing a wide variety of physical and social geographic factors previously identified in the literature. Although the causal evidence supporting the creation and modification of the geographic environment to resolve the lack of recreational spaces was limited, positive effects were consistent.

Within the different approaches undertaken to create recreational spaces, the temporary provision of “Play Streets” bears recognition as an innovative approach that effectively addressed structural barriers and recreational space limitations. Three studies published after 2015 examined the physical activity effects from “Play Streets” in Colombia, San Francisco, and Belgium.^[23,32,36]^ Although there was no overlap in the evaluation designs implemented by the “Play Streets” assessments, it can be noted that these interventions were associated with positive physical activity outcomes in children^[23,32]^ and adults.^[36]^ Moreover, these approaches proved to be successful despite documented differences across countries in the geographic factors associated with physical activity.^[42]^ Besides increasing physical activity, these temporary programs offered opportunities for community engagement and social interaction with potential benefits to social and mental health.^[23]^ A key recommendation for researchers is to further document the physical and social health benefits and challenges of the “Play Streets” interventions using an international framework that takes into account cultural, economic, and geographic differences across countries. The work by the International Physical Activity and the Environment Network study^[42]^ can guide this analytical framework.

We found that most approaches that recognize the influence of geographical environments are not limited to physical factors. Social factors played a key role in the development of sustainable and effective interventions. Supporters of the socioecological perspective^[1,14,43]^ highlight the importance of assessing both, physical and social geographic factors. In a literature review, Mackenbach et al^[12]^ found perceptions of the geographic environments to be a key predictor of physical activity, particularly in disadvantaged neighborhoods. Similarly, in a systematic review of multilevel studies that examined the effects of the interaction between neighborhood socioeconomic status and geographic factors on individual health, Schüle and Bolte^[44]^ found that neighborhood socioeconomic status can mediate the association between built-in environments, and residents’ health. This review identified a variety of social factors such as social support, crime and safety, etc., which, in addition to neighborhood socioeconomic status influenced physical activity uptake (see Tables 2 and 3). Studies reviewed showed that both physical and social geographic factors independently and together not only influence physical activity but also sedentary behaviors.^[26,41–46]^

Poor neighborhoods often encompass unstable and unsafe housing, and more frequent exposure to neighborhood violence and trauma,^[20,22,47]^ which are social barriers to physical activity.^[20,22]^ Identifying the location of social barriers to physical activity can improve physical activity interventions and inspire solutions to overcome social challenges and produce sustainable behavior changes in disadvantaged neighborhoods. In the interventions reviewed, findings from formative research guided tailored approaches that considered the resources and barriers in the environment.^[20,21,23–27,30,32,38]^ A noteworthy example of the benefits of geographic formative research was the implementation of police-patrolled walks to address safety concerns in underserved communities.^[22,38]^ The proper identification of the multiple complex factors influencing physical activity allows public health practitioners to not only track changes, but to better understand *how* interventions bring about the physical activity outcomes. Future research should consider investigating the role of both, perceived and objective, environmental physical and social factors in physical activity interventions, particularly during formative research. Documenting how interventions modified the physical activity of participants is as important as documenting the physical activity outcomes.

A limited number of the physical activity interventions reviewed employed a qualitative methodology. Only 21% of the interventions implemented qualitative assessments.^[24,25,27,33]^ The use of qualitative approaches is strongly recommended in physical activity interventions to better understand the influence of local perspectives in physical activity outcomes. City planners, public health practitioners, and community groups planning to implement physical activity interventions should consider qualitative approaches to improve cultural competence and identify the role of attributes in the geographic environment. The lack of resources that may hinder the implementations of qualitative assessment to examine the effects of physical activity promotion with scientific rigor can be alleviated by seeking partnerships with academic researchers. Academics in turn can benefit from prospective natural experiments.

Twelve of the 13 studies with published outcomes reported positive physical activity improvements, suggesting that considerations of the role of geographical factors in the promotion of physical activity positively influenced behavioral changes. Yet, to further advance the field, methodological challenges need to be addressed to allow comparisons across intervention studies.

Findings also present evidence of the risks of increasing health inequalities by implementing physical activity interventions that fail to reach the most disadvantaged populations.^[28,29]^ It has been documented that interventions are most successful among the most affluent and most active.^[29]^ Whenever physical activity interventions do not intentionally promote equity, positive outcomes could also mean these programs are increasing the current physical activity gap and introducing greater challenges to health equity. In this review, the negative effect of remoteness affected the most disadvantaged more severely.^[28]^ In national programs yielding positive results, provision of services was limited in areas of greater need.^[29]^

Studies utilize a broad range of strategies and approaches to use geographic information in physical activity interventions. The variety of physical activity interventions reviewed highlights the need to examine how geographic contexts introduce physical and social barriers and opportunities for behavior change. Geographic factors were shown to influence the effects and potential sustainability of physical activity interventions. The challenges of implementing and evaluating temporospatial variables within health contexts may be responsible for the lack of evidence on the uses and impact of considering geographic environmental factors. This review summarizes 19 studies that collected geographic information in the design, implementation, or evaluation of interventions that promoted physical activity. It is possible that the intricate processes associated with implementing quality physical activity interventions and perceptions of the inaccessibility of software such as GIS played a role in the scarcity of intervention studies identified. Notably, the publication of a third of these interventions (36%) within the last 15 months of our review hints the emergence of a trend that favors socioecological approaches and transdisciplinary research expansion to integrate geographic information. This paradigm-shift previously observed in the cross-sectional literature^[3]^ is responding to the need to foster transdisciplinary collaborations that can develop innovative social, behavioral, and biological solutions to address the worldwide obesity prevalence.

### Gaps in the literature

Findings should be interpreted with caution given the gaps in the literature and the limitations in our review. Within the literature, a key limitation was the scarce number of articles that satisfied our inclusion criteria. To date, evidence of changes in physical activity following improvements in geographical environments is also insufficient. In addition, it is important to address the lack of consistency in measurements of physical activity and geographic environments, which precluded the elaboration of a meta-analyses. More studies that rely on objective measurements of both physical activity and the environment are needed. As a result of the limitations in the literature, we cannot conclusively determine whether measuring or modifying physical, social factors or both, influences changes in physical activity. Rigorous randomized-control trials are needed to reach these conclusions. In addition to rigorous randomized-control trials, evaluations that rely on natural experiments are also recommended. Moving forward, researchers should consider capitalizing in the increasing interest for developing physical-activity-friendly communities to examine whether changing the geographic environment yields improvements in physical activity. Another important gap is to evaluate changes in physical activity that may be attributable to the gentrification of poor neighborhoods across the populations’ social gradient. It has been suggested that without explicit measurement to limit disparities, physical activity interventions may exacerbate difference by benefiting the previously active populations.^[29]^ Disparities that associated with physical activity interventions merit further investigation.

### Limitations of this review

Although this systematic review followed a rigorous protocol, there are important limitations in this review worth recognizing. Our findings are limited by the lack of validity in the self-reported physical activity data. Self-reported measurements of physical activity often differed greatly from objective measurements.^[48]^ Without objective measurements of physical activity or validation assessments that identify and address misreporting issues in self-reported physical activity data, findings linking physical activity improvements to geographical factors must be interpreted with caution.

Although the integration of international studies can enhance our review, the inclusion of studies from multiple countries also introduces limitations. The international variability in built-in environments and the differences in cultural norms that may influence self-reports of physical activity must be recognized. Considering the studies reviewed do not enable cross-cultural comparisons, corresponding multinational conclusions cannot introduce. Also, we limited our search to publications available in English. The overwhelming majority of studies included from developed countries may be attributable to this limitation, and not to the lack of interventions being conducted in middle- and lower-income countries.

This review focused on primary research articles. It is possible that the exclusion of editorials, letters, commentaries, and policy briefs could have hindered the inclusion of physical activity intervention studies that implemented geographic information and were not also reported as primary research.

Different strategies, such as the collaboration of multiple reviewers, and the quality review that focus on mostly objective assessments were implemented to limit the reviewer biases, yet recognizing lower quality papers can introduce reviewer bias despite our efforts. Finally, given the dearth of long-term data, it is plausible the physical activity improvements documented may have resulted from a novelty effect.

## Conclusions

Every place introduces barriers and opportunities for physical activity. The challenge of designing sustainable physical activity programs that encourage populations to be physically active, and that take advantage of the built in resources, inherently requires the use of geographic information. Evidence showed that supporting the creation and modification of the environment to address geographic barriers, specifically the availability of, and proximity to recreational spaces is associated with improvements in physical activity. These findings underscore the importance of using geographic information to identify the sources and location of physical and social factors in the environment that can influence physical activity uptake. However, more research is needed to identify the physical and social geographic factors that consistently influence changes in physical activity, and to determine if causal associations exist. Public health policy makers can benefit from the wide range of opportunities to measure, analyze, and modify physical and social geographic factors introduced by the intervention studies reviewed to promote long-lasting changes and physical active communities.

## Acknowledgments

The authors would like to thank Kevin E. Markes and Renee A. Pond for their assistance with this study.

## Supplementary Material
