# Supplementary material for: Use of Geographic Information Systems in Physical Activity Interventions: a Systematic Review
Source: Prog Prev Med (N Y). 2019 Jun 14;4(2):e0022. doi: 10.1097/pp9.0000000000000022 (PMC6716576; doi:10.1097/pp9.0000000000000022)
Supplement: Supplementary file 1 [file ppm-4-e0022-s001.docx]

**S1 Table. Search terms and operators used to conduct the search.**

|  | **Geographic Information Terms** | **Health/Wellness interventions** | **Physical Activity** |
| --- | --- | --- | --- |
| 1 | GIS | Intervention | Physical Activity |
| 2 | Geographic information | Interventions | Physical Activities |
| 3 | Geographic information system | Program | Exercise |
| 4 | Geographic information systems | Programs | Exercise [MeSH] |
| 5 | Geographic information system [MeSH] | Prevention | Recreation |
| 6 | Geographic mapping | Preventative | Recreation [MeSH] |
| 7 | Geographic mapping [MeSH] | RCT | Sport |
| 8 | Spatial analysis | Randomized control trial | Sports [MeSH] |
| 9 | Spatial analysis [MeSH] | Health education |  |
| 10 | Arcgis | Multimedia |  |
| 11 |  | Case-control |  |
| 12 |  | Case control |  |
| 13 |  | Intervention studies [MeSH] |  |
| 14 |  | Health promotion [MeSH] |  |
| 15 |  | Health education [MeSH] |  |
| 16 |  | Case-control studies [MeSH] |  |
| 17 |  | Behavior change |  |
| 18 |  | Behavioral change |  |
| 19 |  | CBPR |  |
| 20 |  | Community Based Participatory Research |  |
| 21 |  | Community health |  |
| 22 |  | Public health |  |
| 23 |  | Wellness |  |
| 24 |  | Wellbeing |  |
| 25 |  | Health promotion |  |
| 26 |  | Health promotions |  |
| 27 |  | Health campaign |  |
| 28 |  | Health campaigns |  |

**Pubmed and PsycInfo search algorithm:**

"gis" OR "geographic information" OR "geographic information system" OR "geographic information systems" OR "geographic information systems"[MeSH] OR "geographic mapping" OR "geographic mapping"[MeSH] OR "spatial analysis" OR "spatial analysis"[MeSH] OR "arcgis" AND ("Intervention" OR "Interventions" OR "Program" OR "Programs" OR "Prevention" OR "Preventative" OR "RCT" OR "Randomized control trial" OR "Health education" OR "Multimedia" OR "Case-control" OR "Case control" OR "Intervention studies"[MeSH] OR "Health promotion"[MeSH] OR "Health education"[MeSH] OR "Case-control studies"[MeSH] OR "Behavior change" OR "Behavioral change" OR "CBPR" OR "Community Based Participatory Research") OR ("Community health" OR "Public health" OR "Wellness" OR "Wellbeing" OR "Health promotion" OR "Health promotions" OR "Health campaign" OR "Health campaigns") AND ("Physical Activity" OR "Physical Activities" OR "Exercise" OR "Exercise"[MeSH] OR "Recreation" OR "Recreation"[MeSH] AND "Sport" OR "Sports" OR "Sports"[MeSH]) AND (("2000/01/01"[PDat] : "3000/12/31"[PDat]) AND Humans[Mesh])

**Cochrane search algorithm:**

"gis" OR "geographic information" OR "geographic information system" OR "geographic information systems" OR "geographic information systems" (MeSH) OR "geographic mapping" OR "geographic mapping" (MeSH) OR "spatial analysis" OR "spatial analysis" (MeSH) OR "arcgis" **AND** (“Intervention” OR “Interventions” OR “Program” OR “Programs” OR “Prevention” OR “Preventative” OR “RCT” OR “Randomized control trial” OR “Health education” OR “Multimedia” OR “Case-control” OR “Case control” OR “Intervention studies” (MeSH) OR “Health promotion” (MeSH) OR “Health education” (MeSH) OR “Case-control studies” (MeSH) OR “Behavior change” OR “Behavioral change” OR “CBPR” OR “Community Based Participatory Research”) OR (“Community health” OR “Public health” OR “Wellness” OR “Wellbeing” OR “Health promotion” OR “Health promotions” OR “Health campaign” OR “Health campaigns”) **AND** (“Physical Activity” OR “Physical Activities” OR “Exercise” OR “Exercise” (MeSH) OR “Recreation” OR “Recreation” (MeSH) “Sport” OR “Sports” OR “Sports” (MeSH))
